# Generalising Ward’s Method for Use with Manhattan Distances

**DOI:** 10.1371/journal.pone.0168288

**Published:** 2017-01-13

**Authors:** Trudie Strauss, Michael Johan von Maltitz

**Affiliations:** Department of Mathematical Statistics and Actuarial Science, University of the Free State, Bloemfontein, South Africa; Banner Alzheimer’s Institute, UNITED STATES

## Abstract

The claim that Ward’s linkage algorithm in hierarchical clustering is limited to use with Euclidean distances is investigated. In this paper, Ward’s clustering algorithm is generalised to use with *l*_1_ norm or Manhattan distances. We argue that the generalisation of Ward’s linkage method to incorporate Manhattan distances is theoretically sound and provide an example of where this method outperforms the method using Euclidean distances. As an application, we perform statistical analyses on languages using methods normally applied to biology and genetic classification. We aim to quantify differences in character traits between languages and use a statistical language signature based on relative bi-gram (sequence of two letters) frequencies to calculate a distance matrix between 32 Indo-European languages. We then use Ward’s method of hierarchical clustering to classify the languages, using the Euclidean distance and the Manhattan distance. Results obtained from using the different distance metrics are compared to show that the Ward’s algorithm characteristic of minimising intra-cluster variation and maximising inter-cluster variation is not violated when using the Manhattan metric.

## Introduction

The question arises whether Ward’s linkage algorithm, in hierarchical clustering, can be used in combination with Manhattan distances. Authors like [1, 2] and [3] argue that Ward’s linkage algorithm is limited to use with Euclidean distances, and [4] claim that Ward’s linkage method is “based on the Euclidean distance” (see p. 2 of [4]). This is also echoed in the manuals of some software packages, such as [5] and the fastcluster package for R [6].

Regardless of this definition of Ward’s linkage algorithm, there have been cases where Ward’s linkage algorithm was used with Manhattan distances. [7–10] provide examples of where Ward’s linkage is used with Manhattan distances.

This paper aims to validate that it is indeed possible to use Manhattan distances in Ward’s linkage, in two ways. Firstly, we aim to show theoretically that using the Manhattan metric with Ward’s linkage does not violate the criteria determining the suitability of clustering algorithms and secondly, in the application, we provide an example of where the method using Manhattan distances outperforms a method using Euclidean distances. In Section 1 a background of the study is presented. An overview is given of distance measures and hierarchical clustering methods, focussing on Ward’s method, as well as the views on the use of some non-Euclidean distances with Ward’s linkage. Section 2 discusses the generalisation of Ward’s linkage by using an objective function that accommodates Manhattan distances. In Section 3 an application is introduced where languages are clustered hierarchically with Ward’s linkage and Manhattan distances. Results from this clustering method are compared to results from using the Euclidean distances.

## 1 Background

### 1.1 Distance Measures

Let **a** and **b** be defined as two vectors, each with length *p*. We consider the Minkowski distance suggested on p. 453 in [2] defined in vector space *R*^*p*^:

$$\begin{array}{c} D_{Minkowski}\left(a,b\right)={\left[\sum_{i=1}^{p}{\left|a_{i}-b_{i}\right|}^{r}\right]}^{\frac{1}{r}} \end{array}$$ (1)

where *a*_*i*_ represents the *i*^th^ element of the observation vector **a**. The Minkowski distance is the Euclidean distance when *r* = 2 in and the Manhattan or City-block distance when *r* = 1.

If we have a set of *n* vectors, the constructed distance matrix measures the difference between all vector pairs and has the structure *n* rows × *n* columns with zeroes along the diagonal. We are then able to perform cluster analysis using the distance matrix to construct tree diagrams or dendrograms.

### 1.2 Hierarchical Clustering

In cluster analysis observations are grouped into clusters. The optimal grouping is found where similar observations are grouped together as clusters, but the different clusters are separate from one another. [2] explains the agglomerative hierarchical clustering process on p. 455. In this process each observation vector is a seperate cluster initially. We then measure the similarity or distance between the observation vectors by making use of the distance matrix. At each step of the agglomerative hierarchical clustering process the two clusters with the smallest distance between them are merged into a new cluster. An alternative method of hierarchical clustering is the divisive approach, where initially all observations form one cluster that partitions into two clusters at each step of the clustering process. In this paper we consider only the agglomerative approach. The distance between the new cluster and the rest of the clusters is determined by the linkage method. [2] summarises and explains six linkage methods, but for the purposes of this paper, only Ward’s linkage method is considered. Ward [11] suggested that the decision on which pair of clusters to be joined should be based on the optimal value of an objective function. [11] then used the example of least squared error, or minimum variance, as an objective function. Ward’s method, also referred to as the incremental sum of squares method on p. 466 in [2] or Ward’s minimum variance method [12], takes into consideration not only between-cluster distances when forming clusters, but also within-cluster distances. Ward’s method states that, not only should the between-cluster distances be maximised, but the within-cluster distances should also be minimised. The method combines these two properties into one criterion [11].

### 1.3 Ward’s Minimum Variance Method

Ward’s minimum variance method joins the two clusters *A* and *B* that minimise the increase in the sum of squared errors (SSE):

$$\begin{array}{c} I_{AB}=SSE_{AB}-\left(SSE_{A}+SSE_{B}\right) \end{array}$$ (2)

We define the SSE within and between clusters as follows:

$$\begin{array}{ccc} SSE_{A} & = & \sum_{i=1}^{n_{A}}{\left(a_{i}-\bar{a}\right)}^{\prime }\left(a_{i}-\bar{a}\right)\\ SSE_{B} & = & \sum_{i=1}^{n_{B}}{\left(b_{i}-\bar{b}\right)}^{\prime }\left(b_{i}-\bar{b}\right)\\ SSE_{AB} & = & \sum_{i=1}^{n_{AB}}{\left(y_{i}-{\bar{y}}_{AB}\right)}^{\prime }\left(y_{i}-{\bar{y}}_{AB}\right) \end{array}$$ (3)

where:

- **a**_*i*_ represents the *i*^th^ observation vector in cluster *A*, and $\bar{a}$ the centroid of cluster *A*.
- **b**_*i*_ represents the *i*^th^ observation vector in cluster *B*, and $\bar{b}$ the centroid of cluster *B*.
- **y**_*i*_ represents the *i*^th^ observation vector in cluster *AB*, and ${\bar{y}}_{AB}$ the centroid of newly formed cluster *AB*.

In other words, Ward’s minimum variance method calculates the distance between cluster members and the centroid. The centroid of a cluster is defined as the point at which the sum of squared Euclidean distances between the point itself and each other point in the cluster is minimised. [2] also refers to the centroids of the clusters as their mean vectors on p. 463. The centroid of cluster *A* is defined as the sum of all points in *A* divided by the number of points in *A*. [2] states that the objective function to minimise when using Ward’s minimum variance method can also be written as,

$$\begin{array}{c} I_{AB}=\frac{n_{A}n_{B}}{n_{A}+n_{B}}{\left(\bar{a}-\bar{b}\right)}^{\prime }\left(\bar{a}-\bar{b}\right) \end{array}$$ (4)

where $\bar{a}$ and $\bar{b}$ represent the centroids of clusters *A* and *B*, respectively.

Because the objective function is based on the distances between the centroids of the clusters [2, 13] it is necessary to use the squared Euclidean distance as the metric to calculate distances between objects. If the objective function is minimum variance, Ward’s linkage method can only be applied to distance matrices using the squared Euclidean distance metric.

### 1.4 Properties of Ward’s Linkage Method

Three properties are taken into consideration when considering the suitability of a specific clustering algorithm suggested on pp. 471-475 in [2]. These properties are discussed briefly: (i) Lance-Williams Form, (ii) Monotonicity, and (iii) Space Distortion.

#### Lance-Williams Algorithm

[13] suggested an algorithm for updating distances between clusters when new clusters have been formed. The two elements *A* and *B* in a dissimilarity matrix, with the smallest measure of dissimilarity between them, will be clustered together. To find the distance between cluster *AB* and the rest of the elements, [13] suggest the following formula where *d*_*AB*_, *d*_*AC*_ and *d*_*BC*_ are the pairwise distances between clusters *A*, *B* and *C*. If *A* and *B* were to form a new cluster *AB*, the distance between cluster *C* and the new cluster *AB* is denoted as *d*_*C*(*AB*)_. A clustering algorithm belongs to the Lance-Williams family if *d*_*C*(*AB*)_ can be computed recursively by the following formula:

$$\begin{array}{c} d_{C(AB)}=\alpha_{A}d_{CA}+\alpha_{B}d_{CB}+\beta d_{AB}+\gamma |d_{AC}-d_{BC}| \end{array}$$ (5)

where *α*_*A*_, *α*_*B*_, *β* and *γ* are the parameters that together with the distance function *d*_*ij*_ determine the clustering algorithm [12].[14] explains the importance of an algorithm satisfying such a recurrence relation from a computational point of view on p. 331. Should a clustering procedure not satisfy such a recurrence relation, the initial data, as well as the interim data when updating cluster distances, should be retained throughout the entire process. On p. 344 of [15] the author shows that Ward’s method fits the Lance-Williams algorithm, and suggests appropriate parameters. Ward’s minimum variance method satisfies this recurrence relation proposed by [13]. [14, 15] and p. 470 in [2] also provide the values for *α*_*A*_, *α*_*B*_, *β* and *γ* when using Ward’s method of minimum variance:

$$\begin{array}{ccc} \alpha_{A} & = & \frac{n_{A}+n_{C}}{n_{A}+n_{B}+n_{C}}\\ \alpha_{B} & = & \frac{n_{B}+n_{C}}{n_{A}+n_{B}+n_{C}}\\ \beta & = & \frac{-n_{C}}{n_{A}+n_{B}+n_{C}}\\ \gamma & = & 0 \end{array}$$ (6)

where *n*_*i*_ refers to the number of items in cluster *i*, *i* ∈ {*A*, *B*}.

#### Monotonicity

[16] explain on p. 461 that when the monotonicity property of a clustering method holds, each cluster is formed at a “higher dissimilarity level than any one of its components”. Thus, the monotonicity property implies that a cluster cannot join another cluster at a distance that is less than the distance between previously joined clusters before merging. If a clustering method is not monotonic, it is possible that reversals can be encountered in the dendrograms; *i.e.* the resulting graphical interpretations of the clustering could contain crossovers. Monotonic clustering methods are also referred to as “ultrametric” on p. 471 in [2].

[15] provides conditions for the Lance-Williams parameters, which imply the monotonicity property of a certain clustering algorithm:

$$\begin{array}{ccc} & & \alpha_{A}+\alpha_{B}+\beta \geq 1\\ & & min(\alpha_{A},\alpha_{B})\geq 0\\ & & \gamma \geq 0 \end{array}$$ (7)

[15] shows on p. 344 that Ward’s method has the monotonicity property. This is also clear from the Lance-Williams parameters defined for Ward’s linkage method.

#### Space Distortion

When new clusters are formed, the properties of the distances between the original points before clustering do not always stay intact. Clustering algorithms that preserve the characteristics of the distances between the original points are referred to as space-conserving. In contrast, when clustering algorithm changes the properties of distances, the clustering algorithm is space-distorting [13]. A space-distorting clustering algorithm can either be space-contracting or space-dilating.

If the spatial relationship of the distance between original points becomes smaller, *i.e.* observations join existing clusters rather than form new clusters by joining with individual observations, then the system is said to ‘chain’ [13]. In this case, clusters tend to move closer to each other and the clustering algorithm is space-contracting. A space-dilating clustering algorithm is the opposite; an observation joins another individual observation rather than join an already-existing cluster. This means that the spatial relationship becomes larger as clusters form and clusters move further away from each other. [12] mention that either a space-conserving or a space-dilating method is desirable in most applications.

[17] explain that the Lance Williams parameters of a clustering algorithm can be used to determine whether an algorithm is space-conserving, space-dilating, or space-contracting. For an algorithm to be space-conserving, the following conditions regarding the Lance-Williams parameters should hold [17]:

$$\begin{array}{ccc} & & \alpha_{A}+\alpha_{B}=1\\ & & \beta =0\\ & & |\gamma |<\alpha_{A} \end{array}$$ (8)

[1] show that Ward’s clustering algorithm is space-conserving.

### 1.5 Ward’s Linkage and non-Euclidean Distances

The use of Ward’s linkage has typically been limited to the squared Euclidean distance metric as the measure of original distances between observations [2, 3]. This is because the objective function is usually chosen to be the minimum variance, or minimum squared error. The Euclidean distance is related to the measurement of the sum of squared errors; hence the use of this metric when using Ward’s linkage method [1].

The use of Manhattan distances in Ward’s clustering algorithm, however, is rather common. [7], measure the phonetic distance between different dialects in the Dutch language. The authors compare the Euclidean distance measure, the Manhattan distance measure and a measure corresponding to Pearson’s correlation coefficient. Each of these distance measures are used with Ward’s linkage to construct dendrograms, which are compared with a set of gold standard dendrograms, created by expert dialectologists. The Manhattan distance narrowly outperforms the correlation measure and the Euclidean distance measure in their experiments. [7].

[8] investigate the co-occurrance of search terms submitted to the Excite search engine. Co-occurring terms were clustered using Ward’s algorithm. Like [7] the authors also use the Manhattan distance measure and Pearson’s correlation coefficient and find that these two measures provide similar results.

[9] explain that with use of the Manhattan distance, outliers are only slightly emphasised, and use this distance measure with Ward’s linkage method. They confirm that the results from these methods produce better results than other clustering methods for their particular data set. [10] also use Ward’s linkage with Manhattan distances to cluster mine-waste materials.

[18] stated that the Manhattan metric is preferred to the Euclidean distance metric in numeric cladistic studies. The Manhattan distance metric is also preferred for high dimensional and categorical data.

In the next section we provide a mathematical verification for the use of Manhattan distances in Ward’s linkage clustering method.

## 2 A Variation on Ward’s Minimum Variance Method

Authors such as [2] on p. 499, [14] and [15] refer to Ward’s linkage method as the minimum variance method. [11] suggested that the decision on which pair of clusters is to be joined should be based on the optimal value of an objective function. [11] then used the example of least squared error, or minimum variance, as an objective function. It is this example that has become famous as Ward’s method or Ward’s method of minimum variance. Ward’s method is therefore most commonly used with the objective function of minimum variance. If we, however, decide to use the Manhattan distance, we propose using an objective function of minimum absolute deviation.

In the Backround Section 1.3 we discuss the objective function for Ward’s minimum variance method. Below we discuss the objective function used by [12]. We then propose our own objective function. After we have identified an objective function, it is important to know how the distance measure will be updated after each step of clustering. For this, we also discuss the Lance-Williams algorithm for each of the three objective functions.

### 2.1 Previous Extension of Ward’s Method

[12] extend the use of Ward’s method by showing that the same Lance-Williams parameters are applicable even if the objective function is not minimum variance (*i.e.* when the distance metric is not squared Euclidean). They still use the Euclidean metric, but show that these parameters are also applicable to any power *θ* of Euclidean distance where 0 < *θ* < 2, by generalising the objective function. Thus, [12] propose an objective function using the Euclidean distances between all the observations within a cluster and all the observations between clusters. They define a distance, the *e*-distance, between clusters *A* = {**a**_1_…**a**_*n*_*A*__} and *B* = {**b**_1_…**b**_*n*_*B*__} with each vector in *A* and *B* consisting of *p* different values:

$$\begin{array}{c} e\left(A,B\right)=\frac{n_{A}n_{B}}{n_{A}+n_{B}}(\frac{2}{n_{A}n_{B}}\sum_{i=1}^{n_{A}}\sum_{j=1}^{n_{B}}d\left(a_{i},b_{j}\right)-\frac{1}{n_{A}^{2}}\sum_{i=1}^{n_{A}}\sum_{j=1}^{n_{A}}d\left(a_{i},a_{j}\right)-\frac{1}{n_{B}^{2}}\sum_{i=1}^{n_{B}}\sum_{j=1}^{n_{B}}d\left(b_{i},b_{j}\right)) \end{array}$$ (9)

If the objective function is minimum variance, then *d*_*E*2_(**a**_*i*_, **b**_*j*_) denotes the squared Euclidean distance:

$$\begin{array}{c} d_{E2}\left(a_{i},b_{j}\right)={\left(\sqrt{\sum_{l=1}^{p}{\left(a_{il}-b_{jl}\right)}^{2}}\right)}^{2} \end{array}$$ (10)

where *a*_*il*_ represents the *l*^th^ value in observation vector **a**_*i*_ in cluster *A* and $\frac{1}{n_{A}^{2}}\sum_{i=1}^{n_{A}}\sum_{j=1}^{n_{A}}d\left(a_{i},a_{j}\right)$ represents the mean squared error within cluster *A*.

If the objective function is not minimum variance, but rather the function defined in [12], then *d*_*Eθ*_(**a**_*i*_, **b**_*j*_) denotes the Euclidean distance to the power *θ*:

$$\begin{array}{c} d_{E\theta }\left(a_{i},b_{j}\right)={\left(\sqrt{\sum_{l=1}^{p}{\left(a_{il}-b_{jl}\right)}^{2}}\right)}^{\theta } \end{array}$$ (11)

and $\frac{1}{n_{A}^{2}}\sum_{i=1}^{n_{A}}\sum_{j=1}^{n_{A}}d\left(a_{i},a_{j}\right)$ now represents the mean error to the power *θ* within cluster *A*.

[12] show that the Lance-Williams parameters for their objective function, Eq (9) are the same as the parameters for the minimum variance method.

### 2.2 Least Absolute Deviation

[12] define their objective function using the distance between all elements in a cluster and are no longer restricted to the use of the sum of squared errors as objective function. They can, therefore, generalise Ward’s method for the use of any power of Euclidean distance. Since [12] show that using the distance between every single observation is also acceptable in Ward’s clustering algorithm, we generalise the method of [12] further. We now use a *l*_1_ norm distance, the Manhattan metric, to calculate the distances between single observations. Our objective function will be least absolute error. With this objective function, Ward’s method should join the two clusters *A* and *B* that minimise the increase in absolute deviation or absolute error (AE):

$$\begin{array}{c} I_{AB}=AE_{AB}-AE_{A}-AE_{B} \end{array}$$ (12)

We define the within-cluster and between-cluster absolute error as follows:

$$\begin{array}{ccc} AE_{A} & = & \sum_{i=1}^{n_{A}}\sum_{j=1}^{n_{A}}\sum_{l=1}^{p}|a_{il}-a_{jl}|\\ AE_{A} & = & \sum_{i=1}^{n_{B}}\sum_{j=1}^{n_{B}}\sum_{l=1}^{p}|b_{il}-b_{jl}|\\ AE_{AB} & = & \sum_{i=1}^{n_{A}}\sum_{j=1}^{n_{B}}\sum_{l=1}^{p}|a_{il}-b_{jl}| \end{array}$$ (13)

where *a*_*il*_ represents the *l*^th^ value in observation vector **a**_*i*_ in cluster *A*.

We use the *e*-distance *e*(*A*, *B*) that [12] defined between clusters *A* = {**a**_1_…**a**_*n*_*A*__} and *B* = {**b**_1_…**b**_*n*_*B*__} in Eq (9). However, we now have a different objective function and therefore the distance measure *d*_*M*_(**a**_*i*_, **b**_*j*_) is no longer Euclidean, but describes a Manhattan distance:

$$\begin{array}{c} d_{M}\left(a_{i},b_{j}\right)=\sum_{l=1}^{p}|a_{il}-b_{jl}| \end{array}$$ (14)

If we can prove that the distance *e*(*A*, *B*) in Eq (9) suggested by [12] can be used with our measure of *d*_*M*_(**a**_*i*_, **b**_*j*_), we generalise Ward’s method and show that it can be used with Manhattan distances. If this is the case, it follows that the same Lance-Williams parameters are applicable to our objective function. Thus the proof given by [12] will then also hold when we use an objective function based on an L1 distance like the Manhattan distance.

### 2.3 Generalising Ward’s Method: Least Absolute Error Method

Suppose *A* = {**a**_1_…**a**_*n*_*A*__}, *B* = {**b**_1_…**b**_*n*_*B*__} and *C* = {**c**_1_…**c**_*n*_*C*__} are distinct clusters with all the vectors **a**_*i*_, **b**_*i*_ and **c**_*i*_ consisting of *p* elements.

[12] define the constants $\delta_{AA}^{SR}$, $\delta_{BB}^{SR}$ and $\delta_{AB}^{SR}$:

$$\begin{array}{ccc} \delta_{AA}^{SR} & = & \frac{1}{n_{A}^{2}}\sum_{i=1}^{n_{A}}\sum_{j=1}^{n_{A}}d\left(a_{i},a_{j}\right)\\ \delta_{BB}^{SR} & = & \frac{1}{n_{B}^{2}}\sum_{i=1}^{n_{B}}\sum_{j=1}^{n_{B}}d\left(b_{i},b_{j}\right)\\ \delta_{AB}^{SR} & = & \frac{1}{n_{A}n_{B}}\sum_{i=1}^{n_{A}}\sum_{j=1}^{n_{B}}d\left(a_{i},b_{j}\right) \end{array}$$ (15)

where *d* refers to either of the two distances: *d*_*E*2_ defined in Eq (10) or *d*_*Eθ*_ defined in Eq (11).

The constants defined by [12] represent the mean squared error within and between clusters for the minimum variance method, and the mean error to the power *θ* within and between clusters for their Extended Method.

By replacing the distance *d*(**a**_*i*_, **b**_*j*_) in Eq (15) with *d*_*M*_(**a**_*i*_, **b**_*j*_) as defined in Eq (14), we define the mean absolute error within and between clusters. This is exactly what we want to achieve, as our objective function is minimum absolute error. We are therefore able to use our distance measure *d*_*M*_(**a**_*i*_, **b**_*j*_) with similar constants as defined by [12], and we continue to show that the rest of the proof now also holds for our distance metric.

We first define the constants $\delta_{AA}^{M}$, $\delta_{BB}^{M}$ and $\delta_{AB}^{M}$ in terms of *d*_*M*_, our distance measure. The notation of *δ*^*M*^ is henceforth simplified to *δ*.

$$\begin{array}{ccc} \delta_{AA} & = & \frac{1}{n_{A}^{2}}\sum_{i=1}^{n_{A}}\sum_{j=1}^{n_{A}}d_{M}\left(a_{i},a_{j}\right)\\ \delta_{BB} & = & \frac{1}{n_{B}^{2}}\sum_{i=1}^{n_{B}}\sum_{j=1}^{n_{B}}d_{M}\left(b_{i},b_{j}\right)\\ \delta_{AB} & = & \frac{1}{n_{A}n_{B}}\sum_{i=1}^{n_{A}}\sum_{j=1}^{n_{B}}d_{M}\left(a_{i},b_{j}\right) \end{array}$$ (16)

where:

- *δ*_*AA*_ represents the mean absolute deviation *within* cluster *A*: the distance between all the vectors **a**_*i*_ and **a**_*j*_
- *δ*_*BB*_ represents the mean absolute deviation *within* cluster *B*: the distance between all the vectors **b**_*i*_ and **b**_*j*_
- *δ*_*AB*_ represents the mean absolute deviation *between* clusters *A* and *B*: the distance between all the vectors **a**_*i*_ and **b**_*j*_

We note that [12] used the constant $\frac{n_{A}n_{B}}{n_{A}+n_{B}}$, as did [2] on p. 468. Then, similar to the definition of *e*(*A*, *B*) in Eq (9), we define *e*_*M*_(*A*, *B*):

$$\begin{array}{ccc} e_{M}\left(A,B\right) & = & \frac{n_{A}n_{B}}{n_{A}+n_{B}}(\frac{2}{n_{A}n_{B}}\sum_{i=1}^{n_{A}}\sum_{j=1}^{n_{B}}d_{M}\left(a_{i},b_{j}\right)-\frac{1}{n_{A}^{2}}\sum_{i=1}^{n_{A}}\sum_{j=1}^{n_{A}}d_{M}\left(a_{i},a_{j}\right)\\ & & -\frac{1}{n_{B}^{2}}\sum_{i=1}^{n_{B}}\sum_{j=1}^{n_{B}}d_{M}\left(b_{i},b_{j}\right))\\ & = & \frac{n_{A}n_{B}}{n_{A}+n_{B}}(2\delta_{AB}-\delta_{AA}-\delta_{BB}) \end{array}$$ (17)

For another cluster, *C*, similar to *δ*_*AA*_, *δ*_*BB*_ and *δ*_*AB*_ in Eq (16), we define the constants *δ*_*CC*_, *δ*_*AC*_ and *δ*_*CB*_:

$$\begin{array}{ccc} \delta_{CC} & = & \frac{1}{n_{C}^{2}}\sum_{i=1}^{n_{C}}\sum_{j=1}^{n_{C}}d_{M}\left(c_{i},c_{j}\right)\\ \delta_{AC} & = & \frac{1}{n_{A}n_{C}}\sum_{i=1}^{n_{A}}\sum_{j=1}^{n_{C}}d_{M}\left(a_{i},c_{j}\right)\\ \delta_{BC} & = & \frac{1}{n_{B}n_{C}}\sum_{i=1}^{n_{B}}\sum_{j=1}^{n_{C}}d_{M}\left(b_{i},c_{j}\right) \end{array}$$ (18)

where *δ*_*CC*_, *δ*_*AC*_ and *δ*_*CB*_ follow the same convention as *δ*_*AA*_, *δ*_*BB*_ and *δ*_*AB*_ in Eq (16).

Now we have, similar to *e*_*M*_(*A*, *B*) in Eq (17):

$$\begin{array}{ccc} e_{M}\left(A,C\right) & = & \frac{n_{A}n_{C}}{n_{A}+n_{C}}(2\delta_{AC}-\delta_{AA}-\delta_{CC})\\ e_{M}\left(B,C\right) & = & \frac{n_{B}n_{C}}{n_{B}+n_{C}}(2\delta_{BC}-\delta_{BB}-\delta_{CC}) \end{array}$$ (19)

Consider cluster *A* ∪ *B* formed by merging clusters *A* and *B*. We denote *A* ∪ *B* by *K*, and define the constants *δ*_*KC*_ and *δ*_*KK*_, similar to Eq (16):

$$\begin{array}{c} \delta_{KC}=\frac{1}{n_{K}n_{C}}\sum_{i=1}^{n_{K}}\sum_{j=1}^{n_{C}}d_{M}\left(k_{i},c_{j}\right) \end{array}$$ (20)

where *δ*_*KC*_ is the mean absolute deviation between clusters *C* and *A* ∪ *B* (the distance between all the vectors **c**_*j*_ in *C* and all vectors **a**_*i*_ and **b**_*i*_ in *A* ∪ *B*).

Therefore *δ*_*KC*_ should represent the mean absolute deviation between:

- all vectors **a**_*i*_ in *A* ∪ *B* and **c**_*j*_ in *C* (equivalent to all vectors **a**_*i*_ in *A* and **c**_*j*_ in *C*) and
- all vectors **b**_*i*_ in *A* ∪ *B* and **c**_*j*_ in *C* (equivalent to all vectors **b**_*i*_ in *B* and **c**_*j*_ in *C*)

$$\begin{array}{c} ∴\delta_{KC}=\frac{1}{\left(n_{A}+n_{B}\right)n_{C}}(\sum_{i=1}^{n_{A}}\sum_{j=1}^{n_{C}}d_{M}\left(a_{i},c_{j}\right)+\sum_{i=1}^{n_{B}}\sum_{j=1}^{n_{C}}d_{M}\left(b_{i},c_{j}\right)) \end{array}$$ (21)

Similar to Eq (18), the constant *δ*_*KK*_ is defined as:

$$\begin{array}{c} \delta_{KK}=\frac{1}{n_{K}^{2}}\sum_{i=1}^{n_{K}}\sum_{j=1}^{n_{K}}d_{M}\left(k_{i},k_{j}\right) \end{array}$$ (22)

where *δ*_*KK*_ is the mean absolute deviation within cluster *A* ∪ *B* (the distance within all vectors in *A*, *i.e.* **a**_*i*_ and **a**_*j*_ and all within vectors in *B*, *i.e.* **b**_*i*_ and **b**_*j*_, as well as the distance between all vectors in *A* and *B*).

Therefore, *δ*_*KK*_ should represent the mean absolute deviation between:

1. all vectors **a**_*i*_ in *A* and **a**_*j*_ in *A*,
2. all vectors **a**_*i*_ in *A* and **b**_*j*_ in *B*,
3. all vectors **b**_*i*_ in *B* and **a**_*j*_ in *A*, and
4. all vectors **b**_*i*_ in *B* and **b**_*j*_ in *B*.

$$\begin{array}{c} ∴\delta_{KK}=\frac{1}{{\left(n_{A}+n_{B}\right)}^{2}}(\sum_{i=1}^{n_{A}}\sum_{j=1}^{n_{A}}d_{M}\left(a_{i},a_{j}\right)+2\sum_{i=1}^{n_{A}}\sum_{j=1}^{n_{B}}d_{M}\left(a_{i},b_{j}\right)+\sum_{i=1}^{n_{B}}\sum_{j=1}^{n_{B}}d_{M}\left(b_{i},b_{j}\right)) \end{array}$$ (23)

In terms of the original constants, we now have:

$$\begin{array}{ccc} \delta_{KC} & = & \frac{n_{A}n_{C}\delta_{AC}+n_{B}n_{C}\delta_{BC}}{\left(n_{A}+n_{B}\right)n_{C}}\\ \delta_{KK} & = & \frac{n_{A}^{2}\delta_{AA}+2n_{A}n_{B}\delta_{AB}+n_{B}^{2}\delta_{BB}}{{\left(n_{A}+n_{B}\right)}^{2}} \end{array}$$ (24)

We define *e*_*M*_(*K*, *C*) similar to the way we defined *e*_*M*_(*A*, *B*) in Eq (17):

$$\begin{array}{c} e_{M}\left(K,C\right)=\frac{n_{K}n_{C}}{n_{K}+n_{C}}(2\delta_{KC}-\delta_{KK}-\delta_{CC}) \end{array}$$

And write this in terms of *A* and *B*:

$$\begin{array}{ccc} e_{M}\left(A\cup B,C\right) & = & \frac{\left(n_{A}+n_{B}\right)n_{C}}{n_{A}+n_{B}+n_{C}}(\frac{2n_{A}n_{C}\delta_{AC}+2n_{B}n_{C}\delta_{BC}}{\left(n_{A}+n_{B}\right)n_{C}}\\ & & -\frac{n_{A}^{2}\delta_{AA}+2n_{A}n_{B}\delta_{AB}+n_{B}^{2}\delta_{BB}}{{\left(n_{A}+n_{B}\right)}^{2}}-\delta_{CC})\\ & = & \frac{\left(n_{A}+n_{B}\right)n_{C}}{n_{A}+n_{B}+n_{C}}(\frac{2n_{A}n_{C}\delta_{AC}+2n_{B}n_{C}\delta_{BC}}{\left(n_{A}+n_{B}\right)n_{C}})\\ & & -\frac{\left(n_{A}+n_{B}\right)n_{C}}{n_{A}+n_{B}+n_{C}}(\frac{n_{A}^{2}\delta_{AA}+2n_{A}n_{B}\delta_{AB}+n_{B}^{2}\delta_{BB}}{{\left(n_{A}+n_{B}\right)}^{2}})\\ & & -\frac{\left(n_{A}+n_{B}\right)n_{C}}{n_{A}+n_{B}+n_{C}}\delta_{CC} \end{array}$$ (25)

[12] then simplify the second term in Eq (25), using Eq (17):

$$\begin{array}{ccc} e_{M}\left(A,B\right) & = & \frac{n_{A}n_{B}}{n_{A}+n_{B}}\left(2\delta_{AB}-\delta_{AA}-\delta_{BB}\right)\\ ∴2\delta_{AB} & = & \frac{n_{A}+n_{B}}{n_{A}n_{B}}e_{M}\left(A,B\right)+\delta_{AA}-\delta_{BB} \end{array}$$

Now the second term in Eq (25) can be simplified as:

$$\begin{array}{ccc} & & -\frac{\left(n_{A}+n_{B}\right)n_{C}}{n_{A}+n_{B}+n_{C}}(\frac{n_{A}^{2}\delta_{AA}+2n_{A}n_{B}\delta_{AB}+n_{B}^{2}\delta_{BB}}{{\left(n_{A}+n_{B}\right)}^{2}})\\ & & \;=-\frac{n_{C}}{n_{A}+n_{B}+n_{C}}(\frac{n_{A}^{2}\delta_{AA}+n_{A}n_{B}(\frac{n_{A}+n_{B}}{n_{A}n_{B}}e_{M}\left(A,B\right)+\delta_{AA}+\delta_{BB})+n_{B}^{2}\delta_{BB}}{\left(n_{A}+n_{B}\right)})\\ & & \;=-\frac{n_{C}}{n_{A}+n_{B}+n_{C}}(\frac{n_{A}^{2}\delta_{AA}+\left(n_{A}+n_{B}\right)e_{M}\left(A,B\right)+n_{A}n_{B}\delta_{AA}+n_{A}n_{B}\delta_{BB}+n_{B}^{2}\delta_{BB}}{\left(n_{A}+n_{B}\right)})\\ & & \;=-\frac{n_{C}}{n_{A}+n_{B}+n_{C}}(\frac{n_{A}\left(n_{A}+n_{B}\right)\delta_{AA}+\left(n_{A}+n_{B}\right)e_{M}\left(A,B\right)+n_{B}\left(n_{A}+n_{B}\right)\delta_{BB}}{\left(n_{A}+n_{B}\right)})\\ & & \;=\frac{1}{n_{A}+n_{B}+n_{C}}(-n_{A}n_{C}\delta_{AA}-n_{C}e_{M}\left(A,B\right)-n_{B}n_{C}\delta_{BB}) \end{array}$$ (26)

Eq (26) is then substituted into Eq (25):

$$\begin{array}{ccc} e_{M}\left(A\cup B,C\right) & = & \frac{\left(n_{A}+n_{B}\right)n_{C}}{n_{A}+n_{B}+n_{C}}(\frac{2n_{A}n_{C}\delta_{AC}+2n_{B}n_{C}\delta_{BC}}{\left(n_{A}+n_{B}\right)n_{C}})\\ & & +\frac{1}{n_{A}+n_{B}+n_{C}}(-n_{A}n_{C}\delta_{AA}-n_{C}e_{M}\left(A,B\right)-n_{B}n_{C}\delta_{BB})\\ & & -\frac{\left(n_{A}+n_{B}\right)n_{C}}{n_{A}+n_{B}+n_{C}}\delta_{CC}\\ & = & \frac{1}{n_{A}+n_{B}+n_{C}}[(2n_{A}n_{C}\delta_{AC}+2n_{B}n_{C}\delta_{BC})-n_{A}n_{C}\delta_{AA}\\ & & -n_{C}e_{M}\left(A,B\right)-n_{B}n_{C}\delta_{BB}-\left(n_{A}+n_{B}\right)n_{C}\delta_{CC}]\\ & = & \frac{1}{n_{A}+n_{B}+n_{C}}[\left(n_{A}n_{C}\right)\left(2\delta_{AC}-\delta_{AA}-\delta_{CC}\right)\\ & & +\left(n_{B}n_{C}\right)\left(2\delta_{BC}-\delta_{BB}-\delta_{CC}\right)-n_{C}e_{M}\left(A,B\right)] \end{array}$$

$$\begin{array}{ccc} ∴e_{M}\left(A\cup B,C\right) & = & \frac{n_{A}+n_{C}}{n_{A}+n_{B}+n_{C}}e_{M}\left(A,C\right)+\frac{n_{B}+n_{C}}{n_{A}+n_{B}+n_{C}}e_{M}\left(B,C\right)\\ & & -\frac{n_{C}}{n_{A}+n_{B}+n_{C}}e_{M}\left(A,B\right) \end{array}$$ (27)

We have now shown that the proof used by [12] also holds when using *d*_*M*_(**a**_*i*_, **b**_*j*_), a Manhattan distance. The same Lance-Williams parameters as used in Ward’s minimum variance method also apply to the least absolute error version of Ward’s method. We can therefore use Ward’s method with the Manhattan metric.

## 3 Application

In this section we aim to show how groupings of languages, similarities between languages and language traits well known in the field of linguistics can be extracted or independently observed using unsupervised machine learning techniques; that is, to autonomously classify languages without any prior linguistic knowledge, only the assumption that some languages are related to each other. We assume that languages are classified in a similar way to natural organisms, and we are able to classify languages by means of these numerical biological classification methods. Because it is assumed that there exists some sort of hierarchy or evolutionary relationship between the languages, hierarchical clustering algorithm is used to classify the languages.

### 3.1 Literature

[19] observed that languages change over time and follow the same trends as Darwin suggested for biological organisms in terms of evolution and change (see p. 13 in [20]). If we assume that languages can indeed be classified in a similar way to natural organisms, we can classify languages by means of a numerical biological classification system known as numerical taxonomy.

The concept of numerical taxonomy was introduced by Sokal and Sneath in 1963. This approach classifies items, based on their properties or character traits, by using numerical techniques. Numerical taxonomy uses multivariate techniques applied to classification problems [21]. Sokal and Sneath distinguish two types of relationship between organisms: “relationships based on similarity and those based on descent” on p. 95 of [21]. The affinity, or overall similarity between organisms based on specific character traits, is referred to as a phenetic relationship [21] whereas a phylogenetic relationship “aims to show the course of evolution” (see p. 220 in [21]). Phynetic classification is therefore defined as “a system of classification based on the overall similarity of the organisms being classified” [22]. Phyletic or phylogenetic classification, on the other hand, takes into account the evolutionary ancestry of the organisms.

Since we assume that languages are related to each other, and that some languages are evolutionarily closer than others, we use hierarchical clustering to classify the languages.

[23] provide an example of phylogenetic classification of languages. These authors propose a statistical signature based on the frequency of observing bi-grams (adjacent pairs of letters) as explained by [24] and a signature similar to the genetic signature in biology. They use this statistical language signature (SLS) as a quantitative measure to analyse written text, and suggest that the SLS remains more or less constant within languages, but differentiates between languages. Using distance matrices, [23] construct phylogenetic trees of 34 languages. The trees include 33 Indo-European languages and Basque, defined as a language isolate [25] and clearly shown to be so in the way the classification trees are formed. A similar language tree is constructed by [26], where the relative entropy between pairs of texts constitutes the elements of the distance matrix. [26] then apply the Fitch-Margoliash method that uses a weighted least squares method for clustering, to the distance matrix to obtain the language tree [27].

### 3.2 Methodology

Thirty-two Indo-European languages are analysed in this section, with the aim of identifying the phylogenetic relationships between these languages. [23] and [26] suggest the use of translations of the Universal Declaration of Human Rights [28] as corpus. Using the Universal Declaration of Human Rights provides the advantage that the different texts are more or less the same in length. The problem, however, is that borrowed words and words that have exactly the same translation in related languages could bias results when assessing the proximity between languages [23]. For this reason, we aim to expand our analysis to a corpus of non-parallel texts. Many of the non-parallel texts are obtained form newspaper data and are availble from the Leipzig Corpus [29]. For languages not available in the Leipzig Corpus, files are also obtained from the HC Corpus [30], a Bible corpus [31] as well as texts from the Universial Declaration of Human Rights text files (UDHR Corpus) [28]. Table 1 provides an overview of the sources from which the files were obtained. Datasets can be downloaded from the listed sources.

**Table 1:** Language File Sources.

Source	Language(s)
Leipzig Corpus [29]	Afrikaans, Bosnian, Catalan, Corsican, Czech, Danish, Dutch, English, French, Frisian, Calician, German, Icelandic, Irish, Italian, Latvian, Lithuanian, Luxembourgish, Norwegian, Polish, Portuguese, Romanian, Slovak, Slovenian, Spanish, Swedish
HC Corpus [30]	Serbian
Bible Corpus [31]	Scottish, Welsh
UDHR Corpus [28]	Asturian, Breton, Friulian

While all the selected languages use the Latin alphabet, there are different characters or special letters in each language representing different sounds and accents. Whereas [23] mapped each of the accented characters to its closest equivalent in the Latin alphabet, ignoring the linguistic implications, we introduce an alphabet consisting of 65 characters: the 26 letters of the Latin alphabet, blank spaces between characters and 38 special characters found in the languages we analyse. Our extended alphabet is defined in Table 2.

**Table 2:** Table of characters used for analysis.

a	b	c	d	e	f	g	h	i	j	k
l	m	n	o	p	q	r	s	t	u	v
w	x	y	z	_	ä	à	á	â	å	ã
æ	ç	ê	ë	è	é	ì	í	î	ñ	ö
ø	ò	ó	õ	ô	š	ß	ü	ù	ú	û
ý	ž	ś	ź	ð	ż	ł	ć	ą	ę	

#### Statistical Language Signature

The probability of observing a certain character in a linguistic sequence is highly dependent on the previous characters in the sequence as well as the language under consideration [24]. Based on this observation, [23] suggest that for any given language, a statistical language signature (SLS) can be obtained by using bi-grams (adjacent pairs of letters). We are interested in the number of times any given bi-gram is observed in a text. We know that the bi-gram ‘th’ will be observed often in the English language, while a bi-gram such as ‘en’ will be more common in Afrikaans or German. The SLS for each language is based on the number of occurrences of each bi-gram in that specific language. The SLS that we calculate is the relative frequency of the bi-gram in each language. This is one of the methods suggested by [23].

We let *n*_*αβ*_ denote the number of times the bi-gram ‘*αβ*’ is observed in the document. The table consisting of the relative bi-gram frequencies is defined as matrix *RF* with cells:

$$\begin{array}{c} RF\left(\alpha ,\beta \right)=\frac{n_{\alpha \beta }}{\left(n-1\right)} \end{array}$$ (28)

where *n* is the number of characters in the document (including blank spaces)

Matrix *RF* is size 65 × 65. In order to avoid complications when performing cluster analysis, we henceforth describe our data as a set of 32 observations, (for the 32 languages) where each observation is the SLS in vector form. Each observation is a vector of *p* = 65 × 65 = 4225 elements.

[23] investigate the use of the relative bi-gram frequency table as an SLS. They propose that the SLS of a text depends on the language in which it is written and not on its semantic content. However, this also means that languages that share vocabulary, will be clustered togehter. This is especially the case with loanwords. A discussion about loanwords in English is provided in the discussion of the clustering results. Another observation made by [23] is that the SLS is unique to a language. If we assume this is true, we can continue using this quantitative measure in our analyses of languages. We can then quantify the proximity between languages by introducing a concept of distance, appropriate in *R*^4225^.

After each language is assigned an SLS, we determine the distance between the SLS vectors of the languages. We construct a distance or dissimilarity matrix between languages, and then perform a hierarchical clustering method on this matrix. With the results of the hierarchical clustering, we are able to construct a dendrogram. A dendrogram graphically represents the results obtained from a cluster analysis and is similar to the phylogenetic tree constructed by [23]. A dendrogram “shows all the steps in the hierarchical procedure, including the distances at which clusters are merged” (see p. 456 in [2]). Our trees are rooted, as all the languages we use come from the Indo-European family. We perform a cluster analysis, using Ward’s method with the Manhattan metric. The analysis is done in R [32], using the packages stringi [33] for cleaning datasets and cluster [34].

### 3.3 Results

The distance between a pair of languages is obtained by calculating the Manhattan distance between the SLS vectors of those languages. In order to discuss the difference in results when using the Euclidean distance rather than the Manhattan distance, the Euclidean distance matrix is also obtained.

**Fig 1:**
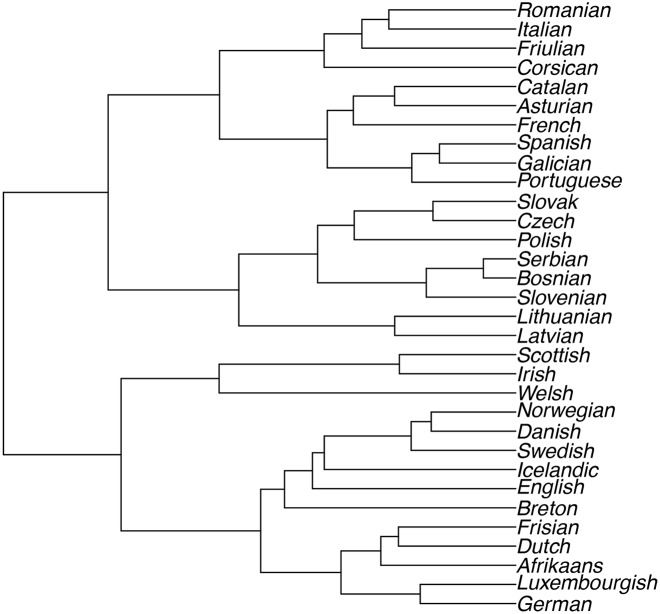
Ward’s Linkage using Euclidean Distances.

**Fig 2:**
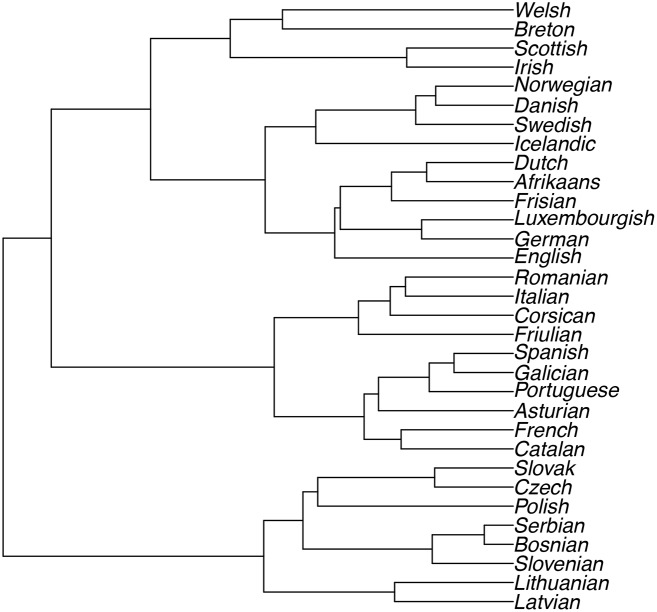
Ward’s Linkage using Manhattan Distances.

We construct dendrograms resulting from the cluster analysis for both objective functions of Ward’s method: minimum variance and least absolute error (*i.e.* using the Euclidean and the Manhattan distance, respectively). Fig 1 shows the results from using Ward’s method with a Euclidean distance matrix and Fig 2 the results from using Ward’s method with a Manhattan distance matrix.

#### Dendrograms

Both dendrograms show the different sub-groups of the Indo-European language family and classify languages from the following subdivisions: Baltic, Slavic (Balto-Slavic), Celtic, Germanic and Romance. The phylogenetic classification of the languages we analyse is as follows:

- Slavic: Bosnian, Serbian, Slovenian, Slovak, Czech, Polish
- Baltic: Lithuanian, Latvian
- Celtic: Breton, Welsh, Scottish, Irish
- Germanic: English, Icelandic, Swedish, Danish, Norwegian, Luxembourgish, Frisian, Dutch, Afrikaans, German
- Romance: French, Italian, Spanish, Asturian, Catalan, Friulian, Galician, Portuguese, Corsican, Romanian

If we consider Fig 1, we note that using the Euclidean distance matrix with Ward’s method results in one language being misclassified into another sub-group of languages. Breton, a Celtic language is clustered with the Germanic languages. English, a West Germanic language, is also misclassified, but still within the Germanic group.

It is worth noting that, although English is classified as a Germanic Language [35, 36], one cannot ignore the influence of Latin and French on the English language. On p. 15 [37] explain that French is a Romance language that was influenced by Latin, and that it is therefore sometimes not possible to discern whether a loanword in English derives from French or Latin. Both of these languages, however, contributed significantly to the English language (see p. 248 of [38]). [38] further explains, on p. 249, in which specific areas French and Latin contributed most to English: The French loanwords in English are typically found in the areas of government and administration (*e.g. Authority, state, liberty, office*), whereas Latin was used as language of the “church, scholarship, and partly of law” (see p. 250 of [38]). Examples of Latin loanwords include *minor, history, individual, explicit*.

Considering the text used to analyse the classification of languages, we notice that the text used by [23], the Universal Declaration of Human Rights, includes a vast amount of these loanwords. Based on the influence of Latin and French, especially in the text that [23] used, we recognise that the algorithm could classify English with the Romance languages rather than the Germanic Languages, as is the case in [23]. Possible misclassification of other languages may also be contributed to the high amount of loanwords in this text, because the SLS depends only on the bi-grams (*i.e.* the lexical aspect of the language) and not on the semantic content [23].

On the other hand, Ward’s clustering used with Manhattan distances, in Fig 2, seems to cluster the languages more or less intuitively from a linguistic and sometimes even geographical point of view. An example of this is that the Nordic Languages or Scandinavian Germanic Languages (Icelandic, Norwegian, Danish and Swedish) are clustered together, before they are joined with the West Germanic (*e.g.* Afrikaans, Dutch and German). Here English is closer to its correct linguistic classification. English belongs with the West Germanic languages as it forms part of the Anglo-Frisian language family [35]. With this method, Breton is also correctly classified as a Celtic language.

Ward’s method yielded the best results when used in conjunction with the Manhattan distance. Using Ward’s method of linkage with the Manhattan distance metric provides us with 5 distinct logical clusters, that also make sense phylogenetically: Baltic Languages, Slavic Languages, Romance Languages, Germanic Languages and Celtic Languages.

### 3.4 Cluster Validation

The discussion above is only based on the visual inspection of the trees in Figs 1 and 2, and is therefore subjective in nature. It is also essential to validate clusters objectively [39]. [39] discuss several techniques of cluster validation, and make the distinction between external and internal measures of cluster validity.

External measures of cluster validity include comparing clustering results with a benchmark or gold standard, while internal measures are based solely on information inherent to the data. These validation techniques “measure how well a given partitioning corresponds to the natural cluster structure of the data” (see p. 3203 of [39]).

**Fig 3:**
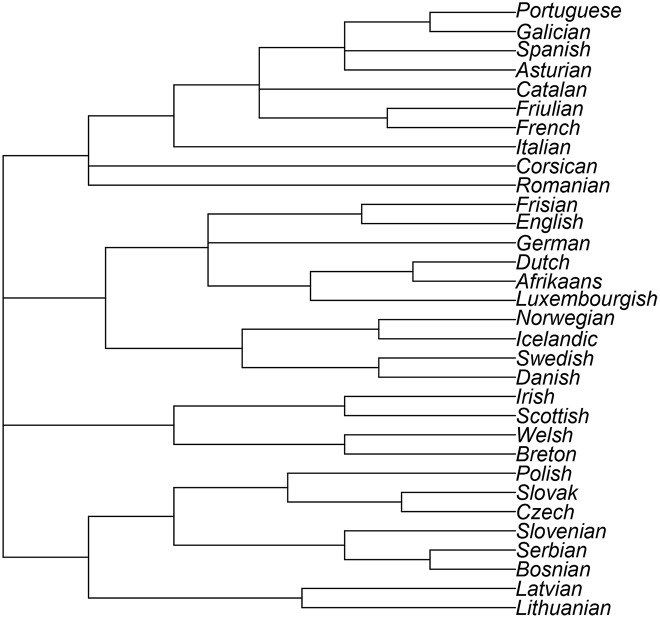
Language Tree with information from Glottolog 2.6.

To compare the figures above with a “gold standard”, a tree is constructed in R [32], to visualise the actual linguistic classifications of these languages. This tree is based on information about language families obtained from Glottolog 2.6 [40] and is shown in Fig 3. We are now able to compare the trees in Figs 1 and 2 with Fig 3. The tree created as benchmark does not necessarily contain the correct branch lengths and weights. This is because it is not possible to quantify the relationship between languages by only considering information on ancestry. For this reason, we suggest using the Robinson-Foulds metric for comparing phylogenetic trees [41].

#### 3.4.1 Robinson-Foulds Distance

[41] suggest a metric to compare different methods of constructing phylogenetic trees. For this comparison, only the structure or topology of the trees is taken into consideration, and not the branch weights [41]. This distance measure between two trees is calculated by “testing edges for matching and counting the unmatched edges” (see p. 146 of [41]). This distance explains which of two trees is closer to a third one, and can be calculated in R [32] using the RF.dist function from the Phylogenetic analysis package, phangorn [42]. The Robinson-Foulds distance between the phylogenetic tree constructed from the Euclidean distance matrix (Fig 1) and the benchmark (Fig 3) is 33. The Robinson-Foulds distance between the phylogenetic tree constructed from the Manhattan distance matrix (Fig 2) and the benchmark (Fig 3) is only 21. Although the Robinson-Foulds metric does not allow us to infer whether this difference in distances is statistically significant [41], we can conclude that using the Manhattan distance matrix for this data set provides results that are closer to the benchmark than those generated by using the Euclidean distance matrix.

#### 3.4.2 Internal Validation Measures

Three of the internal measures discussed by [39] are used in this study. Clusters should be compact and well separated. To validate our clusters, we will consider the following characteristics of the clusters, as mentioned in [39]. The validation is done in R [32], using the Cluster Validation Package clValid [43].

**Compactness and Separation**: If a cluster is compact, it means that homogeneous observations are grouped together, and within-cluster variation is minimised. Where clusters are well separated, the between-cluster variation is maximised. As this is the basis of Ward’s clustering algorithm, we aim to show that using the Manhattan with Ward’s algorithm does not reduce the compactness and separation of the clusters. [39] suggests two measures to evaluate the compactness and separation of clusters, the Dunn Index and the Silhouette Width.

The Dunn Index is defined as the ratio of the smallest between-cluster distance and the largest within-cluster distance. A high value of the Dunn Index would indicate that the smallest between-cluster distance is still larger than the largest within-cluster distance and therefore a high value for this index is desired [39, 43].

The silhouette value of an item measures the degree of confidence in a particular clustering assignment of an individual observation *i* [39, 43], and is calculated as:

$$\begin{array}{c} S\left(i\right)=\frac{b_{i}-a_{i}}{max(b_{i},a_{i})} \end{array}$$ (29)

where *a*_*i*_ denotes the average distance between item *i* and all other items in the same cluster and *b*_*i*_ represents the average distance between item *i* and all items in the closest of the other clusters.

The Silhouette Width is calculated as the average Silhouette value over all observations and yields an answer between −1 and 1. A larger Silhouette Width value is indicative of better clustered observations.

**Connectedness**: The validation test for connectedness attempts to determine to what degree similar items or nearest neighbours, are clustered together [39]. [39] suggest a representative of this connectedness characteristic is the measure of connectivity, counting “the violations of nearest neighbour relationships” (see p. 3204 of [39]). [44] define the connectivity as follows:

If *nn*_*i*(*j*)_ is defined as the *j*^th^ nearest neighbour of item *i*, and *x*_*inn*_*i*(*j*)__ is defined as below:

$$\begin{array}{c} x_{inn_{i(j)}}=\{\begin{array}{c} 0\;\text{if}\;\text{i}\;\text{and}\;\text{j}\;\text{are}\;\text{in}\;\text{the}\;\text{same}\;\text{cluster}\\ \frac{1}{j}\;\text{otherwise} \end{array} \end{array}$$

Then for a data matrix *M* containing *m* rows and *n* columns with a clustering solution *C*_1_, *C*_2_, …*C*_*k*_, the connectivity is defined as:

$$\begin{array}{c} Conn\left(C\right)=\sum_{i=1}^{m}\sum_{j=1}^{n}x_{inn_{i(j)}} \end{array}$$ (30)

The value of the connectivity measure ranges from zero to infinity and a smaller value is desired [44].

Based on these validation techniques, we compare clustering using Euclidean distances with clustering using Manhattan distances. Table 3 provides an overview of the results of these three cluster validation techniques.

**Table 3:** Comparison of Cluster Validation: Euclidean Distance vs. Manhattan distance.

Cluster Characteristic	Validation Measure	Euclidean Distance	Manhattan Distance	Best Result
Compactness and Separation	Silhouette Width	0.2129	0.2571	Manhattan
	Dunn Index	0.5557	0.6246	Manhattan
Connectedness	Connectivity	17.10	16.52	Manhattan

Although the values for the Euclidean and Manhattan distances in Table 3 differ by a small factor, it seems that the Manhattan distance used with Ward’s clustering algorithm yielded better results than using the Euclidean distance in terms of cluster compactness, stability and connectedness. This shows that the important characteristic of Ward’s clustering algorithm, minimising within-cluster variation, and maximising between-cluster variation, is, in fact, enhanced by using the Manhattan distance metric.

## 4 Conclusion

This paper investigated the use of Manhattan distances with Ward’s clustering algorithm. An important component of this research was the generalisation of Ward’s method for use with a *l*_1_ norm distance such as the Manhattan distance. Ward’s method is generalised to include Manhattan distances.

In the application Ward’s clustering algorithm is used to classify a set of Indo-European languages. The results from using Ward’s method with Euclidean distances was compared to the results from using Ward’s method with Manhattan distances. This classification in the form of hierarchical cluster analysis successfully reproduced the phylogenetic classification of the languages. Validation was not only based on comparing the two clusterings with a tree constructed based on the linguistic ancestry, but also on measures such as cluster compactness, separation and connectedness.

The Manhattan distance method yielded the most. The Robinson-Foulds distance between the tree constructed as benchmark and the tree resulting from Ward’s method with the Manhattan distance was less than the distance from the benchmark tree to the tree using Euclidean distances. Using measures like the Dunn Index, Silhouette width and connectivity, it was also shown that intra-cluster distances were minimised and inter-cluster distances were maximised. In our situation, the use of an alternative objective function (in this case least absolute error) with Ward’s function produces more accurate results than Ward’s method with the Euclidean distance metric.

The proposed methodology can not only be applied to other language families beyond Indo-European languages, but also to other languages within this family. Because many other Indo-European languages do not make use of the Latin alphabet, further research could be done on how to compare languages with different alphabets. This could be done by transliterating or using single units of sound from languages with different scripts and map these to their counterparts in the Latin alphabet.

Another possibility for further research is the use of tri-gram frequencies to form the SLS. This would mean having a three-dimensional SLS. The Manhattan metric could still be used in this case, as well as Ward’s method of linkage within the hierarchical clustering process, making this adaptation a natural extension of this research.

Ultimately, we established that Ward’s clustering algorithm can be used in conjunction with Manhattan distances, without the characteristic of minimising within-cluster variation and maximising between-cluster variation being violated, and that for this specific case it produced better results than using Euclidean distances.
